# Impact of postmenopausal osteoporosis on the lives of Omani women and the use of cultural and religious practises to relieve pain: A hermeneutic phenomenological study

**DOI:** 10.1111/hex.13824

**Published:** 2023-07-26

**Authors:** Faiza Al Zadjali, Jane Brooks, Terence W. O'Neill, Emma Stanmore

**Affiliations:** ^1^ School of Health Sciences, Division of Nursing, Midwifery and Social Work University of Manchester Manchester UK; ^2^ Manchester Academic Health Science Centre (MAHSC) Manchester UK; ^3^ Centre for Epidemiology Versus Arthritis University of Manchester Manchester UK; ^4^ Department of Rheumatology Salford Royal NHS Foundation Trust Salford UK; ^5^ NIHR Manchester Biomedical Research Centre Manchester University NHS Foundation Trust Manchester UK

**Keywords:** Arab women, fragility fractures, herbal remedies, pain management, postmenopausal osteoporosis, qualitative study, religious ritual and practice

## Abstract

**Introduction:**

Osteoporosis is a significant clinical and public health concern worldwide. Despite the impact of this condition on women's lives, most studies have focused on its clinical manifestations, drug efficacy, and medical treatment. Furthermore, most studies have been conducted in the West. This study aimed to uncover the personal experiences of postmenopausal Omani women living with osteoporosis.

**Methods:**

In this interpretive phenomenological study, a purposive sample of 15 postmenopausal Omani women with osteoporosis was recruited from primary and secondary care facilities in Muscat, Oman. Semi‐structured one‐to‐one interviews were conducted via Zoom and telephone because of coronavirus disease 2019 restrictions. The interviews were audio‐recorded, and the Ajjawi and Higgs framework was used to analyse the data thematically.

**Results:**

The following key themes were constructed from the interviews: the impact of osteoporosis on religious practices, cultural and social life, and financial status, and the benefits derived from religious and cultural practices and rituals, including Muslim prayer, recitation of Quranic verses, and herbal remedies to cope with osteoporosis‐related pain and suffering.

**Conclusion:**

Osteoporosis and fragility fractures have a significant impact on the religious, cultural, and financial lives of postmenopausal Omani women with osteoporosis. Muslim prayers, recitation of Quranic verses, and herbal remedies are coping strategies for pain in this population.

**Patient or Public Contribution:**

Postmenopausal Omani women with osteoporosis participated in this study through interviews and contributed their lived experiences. Orthopaedic doctors helped recruit patients with postmenopausal osteoporosis.

## 1 INTRODUCTION

Osteoporosis is a major public health condition affecting more than 200 million people worldwide.^1^, ^2^ This skeletal disease is characterised by low bone mass and microarchitectural deterioration of bone tissue, leading to enhanced bone fragility and a consequent increase in fracture risk.^3^ During menopause and ageing, women experience higher osteoclastic activity and gonadal deficiency, leading to low bone mineral density and subsequent bone loss, osteoporosis, and fractures.^4^, ^5^ Hip, wrist, and spine fractures are the most frequent osteoporosis‐related fractures and are associated with significant morbidity, mortality, and healthcare costs.^6^, ^7^, ^8^

Postmenopausal women with fragility fractures may experience severe long‐term pain.^9^ Such pain leads to several functional restrictions, including the inability to perform daily activities, and might cause sleeping difficulties, especially in older adults.^10^ Iolascon et al.^11^ reported that pain levels determine the treatment outcome for these fractures, regardless of their location. Therefore, effective pain management should be used to improve quality of life and can be achieved by incorporating pain interventions congruent with the patients' cultural and religious practices. Healthcare professionals can apply a pain model, such as the biopsychosocial model,^12^ to understand the nature of and effectively relieve their patients' pain.

Osteoporosis research in postmenopausal women has mainly been conducted in Western countries, with limited research in Middle Eastern and predominantly Muslim countries. Previous reports have focused on exploring the experiences of living with long‐term pain,^13^, ^14^, ^15^, ^16^ the experiences of dissatisfaction with the health information provided by healthcare professionals,^14^, ^17^, ^18^, ^19^, ^20^, ^21^ and women not accepting their osteoporosis diagnosis.^13^, ^14^, ^22^, ^23^, ^24^ This study aimed to focus on the experiences of postmenopausal Omani women with osteoporosis and fragility fractures, to understand the meaning of pain and suffering for these women, and to determine the impact of religious and cultural practices on relieving osteoporotic pain. This study seeks to answer the following research questions:

1. What does it mean to be a postmenopausal Omani woman with osteoporosis?
2. What religious and cultural practices do postmenopausal Omani women with osteoporosis use to cope with pain?

## 2 METHODS

### 2.1 Study design

A qualitative research design guided by a hermeneutic (interpretative) phenomenological methodology was used to address the study questions. Heideggerian and Gadamerian phenomenological traditions underpin this study.^25^, ^26^ Hermeneutics is the classical discipline concerned with the art of interpreting texts and is related to the theory and practice of interpretation, the meaning of interpretation, understanding and interpretation (the meaning behind everything), and meaning hidden in everything.^27^ The main aim of hermeneutic phenomenology is to gain an in‐depth understanding of the meaning of lived experiences from an individual's perspective by investigating interactions among individuals and their inhabited historical and cultural contexts.^28^

### 2.2 Recruitment of participants

Fifteen postmenopausal Omani women with osteoporosis were recruited from two different settings in Oman, between October 2020 and April 2021. One setting is home to several specialist clinics, including an orthopaedic clinic, where Omani women with osteoporosis receive routine care and attend follow‐up appointments.^29^ The other is a secondary healthcare hospital in the Muscat region and a tertiary healthcare hospital in Oman. It is the only inpatient healthcare facility that receives complex cases of spinal and hip fractures^29^ and primarily functions as a trauma centre for orthopaedics, neurosurgery, and burns.^29^ The rationale for selecting these settings was to include women with a range of disease severity, including those with and without fractures.

Participants were purposively sampled for the interviews. Postmenopausal Omani women at different stages of osteoporosis, with or without a history of low‐trauma fractures, were included. The mean age of participants was 63 years. One‐third (33.3%) of patients had experienced a fracture in the past. More than half (53%) lacked literacy skills, and a minority (20%) had received a college education. Three of the participants had paid work experience.

Participants were identified by their orthopaedic doctors based on the inclusion criteria. Table 1 shows the inclusion and exclusion criteria, Table 2 shows the participants' characteristics, Table 3 shows the participants' osteoporosis details, and Table 4 shows the definitions of the participants' risk factors.

**Table 1:** Participants' inclusion criteria.

Inclusion criteria	a) Postmenopausal Omani women diagnosed with osteoporosis with or without a history of low trauma fracture, as confirmed by a clinician. b) At least 12 months following the last menses. c) Able and willing to provide informed consent (verbal). d) Willing to be audio‐recorded. e) Able and willing to describe their lived experiences. f) Arabic/Balushi or English speakers.
Exclusion criteria	a) Significant cognitive impairment.

**Table 2:** Participants' characteristics.

Participant code	Age (years)	Marital status	Number of children	Living area	Educational level/language spoken	Comorbidity
P1	62	Married	5	Capital city	Illiterate/Arabic and Baluchi	Hypertension & high cholesterol
P2	50	Married	7	Capital city	Studied up to grade six/Arabic	Hypertension/disc prolapse/osteoarthritis in both knees
P3	66	Married	11	Capital city	Illiterate/Arabic	Hypothyroidism
P4	78	Widow	6	Capital city	Studied Holy Quran school/Baluchi	Heart failure/rheumatoid arthritis/hypertension
P5	63	Married	11	Capital city	Studied up to grade three and Holy Quran school/Arabic	Hypertension/high cholesterol
P6	55	Widow	2	Capital city	Bachelor's degree in social science and philosophy/Arabic	High cholesterol
P7	80	Married	6	Capital city	Illiterate/Arabic	Heart failure/hypothyroidism
P8	80	Widow	4	Urban area	Illiterate/Arabic	High cholesterol
P9	70	Widow	7	Urban area	Illiterate/Arabic	Heart disease
P10	66	Widow	10	Urban area	Illiterate/Arabic	Hypertension/high cholesterol/osteoarthritis in both knees
P11	60	Married	9	Urban area	Illiterate/Arabic	Hypertension
P12	56	Married	8	Urban area	Illiterate/Arabic	Hypertension
P13	46	Married	4	Urban area	Diploma in general nursing programme/Arabic	Asthma
P14	47	Married	6	Urban area	Diploma in education/Arabic	Cushing syndrome
P15	63	Married	14	Capital city	Studied Holy Quran school/Arabic and Baluchi	Hypothyroidism/diabetes mellitus/rheumatoid arthritis

**Table 3:** Participants' osteoporosis details.

Participant code	Previous bone density test	Number of years since diagnosis	Presence of fragility fractures	Site of fractures	Presence of risk factors for osteoporosis	Osteoporosis therapy (oral bisphosphonate/IV/HRT/calcium/vitamin D)
P1	Yes	8 years	No	–	Premature menopause at 45 years old	Oral bisphosphonate/calcium/vitamin D
P2	Yes	5 years	No	–	Premature menopause <45 years old	Oral bisphosphonate/calcium/vitamin D
P3	Yes	10 years	No	–		Bisphosphonate IV/calcium/vitamin D
P4	Yes	12 years	Yes	Vertebra	Low dietary calcium intake	Oral bisphosphonate/calcium/vitamin D
P5	Yes	19 years	No	–	Low dietary calcium intake	IV bisphosphonate/calcium/vitamin D
P6	Yes	5 years	Yes	Lt. hand	Family history of osteoporosis (mother)	Calcium/vitamin D
P7	Yes	6 years	Yes	Vertebra		Oral bisphosphonate/calcium/vitamin D
P8	Yes	8 years	No	–		Oral bisphosphonate/calcium/vitamin D
P9	Yes	2 years	No	–		Oral bisphosphonate/calcium/vitamin D
P10	Yes	10 years	No	–	Low dietary calcium intake	Oral bisphosphonate/calcium/vitamin D
P11	Yes	14 years	No	–		Oral bisphosphonate/calcium/vitamin D
P12	Yes	12 years	Yes	Vertebra/pelvic and legs		Oral bisphosphonate/calcium/vitamin D
P13	Yes	2 years	Yes	Hip	Premature menopause <45 years old	IV bisphosphonate/calcium/vitamin D
P14	Yes	2 years	No	–	Premature menopause <45 years old	IV bisphosphonate/calcium/vitamin D
P15	Yes	12 years	No	–	Family history of osteoporosis (mother) and low dietary calcium intake	Oral bisphosphonate/calcium/vitamin D

Abbreviations: IV, intravenous therapy; HRT, hormonal replacement therapy.

**Table 4:** Definition of the list of risk factors among participants.

List of risk factors	Definition
Family history	Family history of hip fracture and osteoporosis (parents/siblings only).
Low dietary calcium intake	Not taking milk and its products at all.
Premature menopause	Age <45.
Steroid use	>3 months.

### 2.3 Data collection

Eligible participants were asked to participate at their convenience in a 45–60 min semi‐structured online interview due to coronavirus disease 2019 (COVID‐19) pandemic restrictions. Twelve participants agreed to be interviewed through an audio zoom call, and three agreed to be interviewed by phone. All women who agreed to Zoom calls declined to use the video interface and chose audio only. This may reflect the conservative culture among Omani women. All interviews were conducted by the first author, an Omani woman. Verbal consent was obtained from all the participants. All data were kept confidential, and the recordings were encrypted and saved in a secure storage facility at the University of Manchester. Participants were advised to withdraw from the study without prejudice at any time. The researcher who conducted the interviews was not involved in providing care to the participants, and the researcher's professional background as a nurse was not disclosed to the participants.

Since sharing the same native language increased awareness of the meanings of certain phrases related to the sociocultural context, topic guide questions were constructed in English and translated into Arabic and *Balushi* by the first author, a fluent Arabic and *Balushi* speaker. To ensure the authenticity of the translation of interview transcripts, the translation and back‐translation of the two interview transcripts (one in Arabic and the other in *Balushi*) were checked for accuracy by a professional translation agency in Oman, and no errors were found. Clark et al.^30^ highlight that having the same researcher work on transcribing, translating, and interpreting the data increased the researcher's familiarity with and sensitivity to the data set, which, in turn, enhanced the authenticity of the research findings.

As Arabic is the main language used in Oman,^31^ 12 interviews were conducted in Arabic. However, three interviews were conducted in *Balushi* (*Balochi* [Balochi is one of local language in Oman]) based on the participants' requests. This was in line with the findings of Baumgartner^32^ regarding the use of participants' language to enhance the depth of engagement and mutual understanding. Table 5 presents the details of the translation process.

**Table 5:** Translation process.

Topic guide questions	The process of translation
*Background information*	
Tell me a little bit about yourself. ○ Where are you from? ○ How would you describe yourself as a person? ▪ Age ▪ Sociocultural background ▪ Educational level Tell me a little bit about your family (children). If you are working, could you tell me little bit about your work please? How does your work affect your daily life? What activities do you enjoy? Have you been forced to give anything up since becoming unwell? When you were younger did anyone talk to you about OP? If they had, do you think you would have done anything differently? If so, what? Do you think there are facilitators or barriers in the prevention of OP in women of your age? If so, what are they?	After preparation of the topic guide in English the first author who is a fluent Arabic and Balushi speaker undertook translation of the guide to Arabic and Balushi. After interviewing the participants, the first author back translated these interviews to English. Then she transcribed each interview verbatim and analysed to identify themes. To ensure the authenticity of translation of interview transcripts, the translation and back‐translation of the two interview transcripts (one in Arabic and another one in Balushi) were checked for accuracy by a professional translation agency in Oman and found no errors in these two transcripts.
*Nonmedical help seeking*	
How did you feel when you first felt unwell? For how long had you felt unwell? What did you think then? Whom did you speak to? What help or advice did you try before you went to a doctor? What support did you receive from family or friends? If you sought support from friends or family about your health, how much did you tell them? How did you feel about becoming unwell? What, if anything, did you find most difficult about being unwell and why?	
*Exploring diagnosis of OP*	
Could you tell me about your health problems? Was there a particular incident or trigger that made you go to see a doctor? What did you think you had? What did they tell you about what you had? Have you been given a name for your condition? How long did it take before you were given a name for your condition? Did you have any worries or concerns at this point? What were they?	
*After diagnosis*	
What did you understand about the nature of your illness? What did you know about OP? Had you heard of OP? Did you know anyone with OP? What did you think about the risk factors of OP? Was there anything you were worried about at this point? What was it? Whom did you talk to about your worries? What sort of support did you receive from friends and family? Who was the most supportive and what did they do to support you? Did you manage to relinquish any of your family or work commitments and how did that make you feel?	
*Living with OP*	
Have any changes occurred in your life since being diagnosed with OP? How does OP affect your everyday life? Could you please tell me in which term osteoporosis affected you in taking bath? Could you please tell me in which term osteoporosis affected you in going to toilet? What is a good day like? What is a bad day like? Has it affected your ability to carry out your duties and responsibilities? How do you feel now about having OP? Has it changed your attitude towards life? If yes, then in what way? Do you think your illness has affected people's attitude towards you? If yes, then in what way?	
*Family support*
How would you describe your family members in general and your children in particular? In what ways do your children support you? In what ways does your husband support you?
*Coping*
What would you say you do to cope with your illness? What is most helpful? Who has been the most helpful? Who has been the least helpful? If there are any friends or family members who are not helpful, how do you feel about that? What would you like them to say and do? Have you faced any particular problems/challenges as a person with OP? What are they? How do you cope with daily life in self‐managing the disease? Do you ever feel you cannot cope? Have you had any problems/challenges of treatment? How have you coped with them? What do you think about prayer and religion as coping mechanisms?
*Relationship with health professionals*
How often do you see a health professional? What sort of health professional do you see and are they sympathetic and helpful? What happens when you see them? What has been most helpful to you in your care and treatment? What has been least helpful? Is there anything you would find helpful that is missing in your care?
*Challenges faced with transportation/travelling/waiting time/health education*
Do you face any challenges in relation to travelling or finding transportation from your area to X hospital? Are you facing any difficulties finding someone to bring you to the clinic for your appointment? How do you manage your waiting time before seeing the consultant? Do you have any difficulties understanding your treatment plan? Are the healthcare professionals supportive in terms of educating you about the OP medication, preventive activities, and dietary requirements?
*Concluding question*
Is there anything more you would like to add from your experience and perceptions of living with OP which we have not covered in this interview? Would you like to share it with me?

Abbreviation: OP, osteoporosis.

### 2.4 Data analysis

All interviews were transcribed verbatim using audio recordings. They were then translated from Arabic or *Balushi* to English. Data transcription and translation were performed immediately after each interview. Although software such as NVivo is used to good effect in qualitative studies,^33^ manual analysis was chosen for this study.^34^ The researcher found manual analysis more suited to their way of working, despite the acknowledged effectiveness of NVivo and other software. Manual analysis refers to the process of analysing and examining data or information manually without the use of electronic technology or automated tools.^35^ This involved reviewing the data, organising with codes or categories, and interpreting their meanings and themes to allow full engagement and deep understanding of the data.

In keeping with the methodology adopted in this study, the data analysis methods were developed based on hermeneutic phenomenological principles, and the Ajjawi and Higgs^36^ analysis framework was chosen. The key concepts of both the qualitative methodology and philosophical hermeneutics are addressed in six steps. Three themes were identified: the impact of cultural and religious practices on the participants' lives, their attitudes towards healthcare professionals and health services, and their experiences with and attitudes towards various treatment regimens. Discussions were held between the researcher and the supervisory team to finalise the themes generated in this study. Table 6 presents an integrative thematic analysis of philosophical hermeneutic tenets.^36^, ^37^

**Table 6:** Integrative of thematic and philosophical tenets.

Ajjawi and Higgs^36^ interpretive analysis steps	Braun and Clarke^37^ thematic analysis steps	Philosophical hermeneutic principles
Stage 1: Immersion	Familiarisation with the data.	Preunderstanding. Use of reflexive diary. Fusions of horizons. Preliminary interpretation of the text to facilitate coding.
Stage 2: Understanding	Code generation.	Use of hermeneutic circle. Codes represented participant's horizons.
Stage 3: Abstraction	Categories generation. Subcategories generation. Categories and subcategories were grouped manually into subthemes.	Subthemes represented my horizons.
Stage 4: Synthesis and theme development	Grouping of subthemes into themes. Development of themes.	Use of hermeneutic circle.
Stage 5: Illumination and illustration of phenomena	Naming and defining themes. Linking the literature to the themes identified above.	Reconstructing interpretations into stories. Completion of the hermeneutic circle.
Stage 6: Integration and critique	Producing the report.	

## 3 RESULTS

### 3.1 Impact of osteoporosis on women's lives

#### 3.1.1 Religious life

The participants cited the impact of osteoporosis and fragility fractures on religious practices, prayers, and the recitation of Quranic verses.

Ever since I had fractures in my vertebra, I have not been able to pray on the floor, so I pray while sitting on a chair. I also read the Holy Quran while sitting on a chair, whereas previously, I read it after praying on a prayer mat. (P4)

Since I cannot pray on prayer mats because of pain, I pray while sitting on a chair and recite Quranic verses. (P1)

Data analysis suggests the importance of religious practices for ageing people and how health conditions can affect their ability to fully participate in these practices.

#### 3.1.2 Cultural and social life

The participants expressed the impact of osteoporosis and fragility fractures on their cultural and social practices.

My children and grandchildren gather at my house every Friday from the afternoon until night; my daughters prepare lunch and dinner, and we all gather to have meals. Women eat together from one big plate, and men eat together from another. I cannot eat with them because all of them eat from one plate on the floor, and I cannot sit on the floor because of pain. I sit in a chair and eat my meals separately. (P10)

Since I developed fractures and fragility in my spine, I have been confined to my home, and I cannot walk properly because of severe pain. (P4)

The findings of the data analysis indicate that osteoporosis and fragility fractures have destructive influences on the societal, cultural, and religious aspects of the participants' lives.

The support from participants' daughters was evident.

I have severe osteoporosis and am unable to do household chores. My children hired a helper who did all the household chores except cooking. I have two daughters: one prepares breakfast and lunch, and the other prepares dinner on weekdays. On weekends, both prepared three meals, because one of my daughters works in the private sector and can only prepare dinner on weekdays. Before I developed osteoporosis, I performed all my household chores, and I was a typical *Bedu* woman, as I had camels, goats, and cows. I took care of them, and now I depend on the helpers and my daughters. I can only supervise my daughters and the helpers. I have three grandchildren with me at home, but one of my daughters goes to work while the other daughter takes care of them. I just supervise them. (P8)

According to the data analysis, participants reported a reliance on female family members, especially daughters, which is common in the Omani culture. The absence of these women from the support network can have a significant impact on their families, particularly large, extended families. In Omani culture, daughters are expected to take care of their ageing parents, providing emotional support, and may also be responsible for household duties and childcare. The ramifications of one or more daughters leaving the support network for paid work or on marriage can leave a gap in the family support system and lead to increased stress and responsibility for remaining family members.

Some participants reported how osteoporosis affected their role as housewives.

My husband does not like to eat food prepared by the housekeeper. However, I have severe generalised body pain and cannot stand for a long time. Therefore, I have to sit on a chair to cut my vegetables. Despite experiencing severe pain, I had to prepare my meals. My daughters go to work, and I do not have any other choices. My husband wants lunch ready at 1:00 pm. (P5)

The data analysis suggests that gender roles and expectations in Omani society are greatly influenced by its historical and cultural background, as evidenced by the experiences shared by multiple women.

#### 3.1.3 Financial impact

Despite traditional attitudes towards women working outside the home for financial gain, such edicts were clearly circumvented in some circumstances. Some Omani women were obligated to earn money, but this was recast as domestic work to prevent the contravention of cultural norms and expectations. In these instances, the economic impact of living with osteoporosis is also evident.

My husband was an elderly man who did not work. I worked before I developed osteoporosis. I sewed *abayas* (lightweight covers for all gowns worn over standard clothes) and sold them. I also made and sold traditional beauty products for brides. I am famous in my community for this line of business. In addition, I washed dead women in preparation for burial, and people called me when their female relatives or neighbours died. My financial status is excellent, and I am self‐sufficient. After developing osteoporosis, I could not do any of the work I enjoyed and became bored and dependent on my children and grandchildren. (P4)

Before I developed osteoporosis, I sewed traditional clothes for women and performed face and eyebrow threading. I earned a lot of money, and my financial status was good. I cannot do all of this work after being diagnosed because I have severe pain in my hands and back. (P1)

I am a *Bedu* woman, and the Bedouin people must have goats in their houses. You know, before I developed osteoporosis, I cared for camels and cows. I also trade goats, camels, and cows. I have been a wife for 20 years, and my children were young when their father died. This business helped me raise my children. I have done this business for a long time, and now I feel that I am very deficient because I have to stop running my business. You know that, among the Bedouin tribes, it is shameful if you do not do your traditional work. (P8)

Recent changes in Omani society, particularly regarding women's rights and participation in the workforce, have meant that women's contributions to the economy of the family and the country are now openly promulgated. This is evidenced by the three younger participants who had high educational levels and professions. Table 7 shows the differences in age, educational level, and employment status between younger and older participants.

**Table 7:** The differences between the demographic variables and employment status between the younger and older participants.

Participant code	Age (years)	Educational level	Employment status
P1	62	Illiterate	Housewife
P2	50	Studied up to grade six	Housewife
P3	66	Illiterate	Housewife
P4	78	Studied Holy Quran school	Housewife
P5	63	Studied up to grade three and Holy Quran school	Housewife
P6	55	Bachelor's degree in social science and philosophy	Social worker
P7	80	Illiterate	Housewife
P8	80	Illiterate	Housewife
P9	70	Illiterate	Housewife
P10	66	Illiterate	Housewife
P11	60	Illiterate	Housewife
P12	56	Illiterate	Housewife
P13	46	Diploma in general nursing programme	Head nurse
P14	47	Diploma in education	Teacher
P15	63	Studied Holy Quran school	Housewife

### 3.2 Religious and cultural rituals and practises as coping mechanisms

The participants cited the use of culturally related interventions to relieve osteoporosis‐related pain, including the value of cultural gatherings with their relatives and neighbours. Despite the participants' difficulties in socialising at cultural gatherings, they valued attending these events, which acted as distractors for their pain and suffering.

Osteoporosis does not affect my social life, because I am a very social and love‐visiting person. I have severe back and knee pain, but I attend wedding parties and regularly visit my relatives. I want to ignore my pain and these cultural gatherings have helped me to do so. (P2)

I have good relationships with my neighbours and relatives. I visit them weekly. Although I have a fracture in my back, I attend close relatives' and neighbours' weddings and gatherings. I have a daily evening routine of sharing Omani coffee with my neighbours. Having coffee with my neighbours and chatting with them about our lives helped me forget the pain of my fragility fractures. (P7)

Participants cited the use of Muslim prayers and recitation, or listening to the Holy Quran, as coping mechanisms for pain and suffering.

When I read the Holy Quran, I forget about my pain. The Holy Quran provides *sakina* (Sakina means peace and tranquility). I believe that the Holy Quran is powerful medicine. I read the Holy Quran every night. Because of these effects, I do not feel severe pain in my hands or back. I believe that the more pain I suffer, the more *ajer* (Ajer means reward from Allah) I receive from *Allah* (Allah is the God of Muslims). This is what we obtained from the Holy Quran. (P1)

You know, one cannot feel that they are praying properly unless they make *soojod* (Soojod means prostration) because it provides *sakina*. When I *sujud* (Sujud is the act of low bowing or prostration to God facing the giblah (direction of the Kaaba at Mecca)), I do not feel any pain in my back, but once I am done with my prayers, the pain starts again. I strongly believe that with more pain, I will get *ajer* from *Allah*. (P11)

Although all the participants in this study were culturally Muslim, some considered themselves to be devout Muslims.

I lead a very busy life. I wake up at 3:00 am every morning and start my day reading the Holy Quran and praying. I then continued praying and read the Holy Quran until sunrise. I prayed for and read the Holy Quran for more than 12 h a day. I prayed and read the Holy Quran on a chair now, unlike before when I read the Quran and prayed on a prayer mat. I prefer praying on a prayer mat because I feel like I am praying properly. (P1)

Some participants cited the use of prayers while waiting for appointments with doctors.

As I told you, sometimes I wait for more than 2 h to see the doctor. I do not feel bad about these 2 h because I spend my time in *istighfar* (Istighfar is a type of praying either with finger counting or with the use of tasbiha). I take my *tasbiha* (Tasbiha is a chain Muslims use for praying) in my bag to make *istighfar*. The more *istighfar* you are, the more *ajer* you will receive. You will live a peaceful life. (P1)

I wait for more than 2 h; I effectively use my time. So that I can get *ajer*, I make *istighfar* and other prayers such as prayers for the Prophet Mohammed *sallallahu alayhi wa salaam* (*sallallahu alayhi wa salaam* means may God's prayers and peace be with him). (P5)

### 3.3 Herbal remedies as cultural practice for pain relief

Many participants cited the use of herbal remedies to relieve osteoporosis and fragility pain.

I worked as a head nurse and was admitted to X Hospital because I had fragility fractures and was continuously receiving pain injections, such as tramadol. When I was discharged, I took paracetamol and started to use herbs such as *haba al hamra* (haba al hamra is a type of herbal remedy used to strengthen bones). They have been used in combination with osteoporosis treatment. I received many benefits from this herb, as it relieved my pain and strengthened my bones. We believe that herbal medicines are effective in the treatment of osteoporosis. There is no harm to using herbs; even if you do not get any benefits, they do not do any harm. (P13)

I am an old Bedouin woman. Every night I massage my back, legs, and hands with olive oil. I take *Tefersit* (tefersit is a type of herbal remedy used to strengthen bones and in cases of fracture) every night with milk to relieve my leg and body pain. (P12)

Several participants strongly believed that herbal remedies had no side effects owing to their natural origin.

I used osteoporosis tablets and had severe stomach pain and constipation that I could not tolerate. I stopped taking them and replaced them with an herbal medicine (*tefersit*). Herbal medicines are considered safe and have no adverse effects. (P1)

I experienced severe burning in my stomach because of the calcium tablets I took on alternate days. I replaced them with an herbal medicine (*Tefersit*) because these medicines do not have any side effects. (P2)

Family was a motivating factor for some participants to use herbal remedies.

My daughter supported me a lot, and she always took me to the herbal shop to buy herbal medicine. She encouraged me to take herbal medicines because I felt well when I took them. (P1)

My daughter always takes me to the herbal shop to buy herbal medicines and I am healthy when I take them. (P12)

According to the data analysis, prayer and traditional medicine may serve separate functions in addressing pain; however, they are closely associated with the religious and cultural beliefs of postmenopausal Omani women with osteoporosis. Mindfulness, a connection with Allah, and emotional stability are some of the benefits of prayers. Traditional medicine is viewed as a practical method for managing physical symptoms such as pain relief. By merging these techniques, women may have a comprehensive solution for pain management that coordinates with their cultural values and faith.

## 4 DISCUSSION

Several themes were generated from the data: the impact of osteoporosis on religious life, cultural and social life, financial status, and the use of religious and cultural rituals and practices as coping mechanisms. This study confirmed that osteoporosis and fragility fractures have a significant impact on the daily lives of postmenopausal Omani women with osteoporosis, including practising daily prayers and reciting Quranic verses, their financial status, and feelings of social isolation and a lack of participation in cultural practices. Some women manage their pain and suffering through Muslim religious practices and cultural interventions.

Some participants expressed sadness that they could not perform sujud (prostration) on a prayer mat because of severe pain in their legs, spine, and bodies. Research on alpha brain activity during prostration has shown increased amplitudes in the parietal and occipital regions, suggestive of parasympathetic elevation, thus indicating a state of relaxation.^38^, ^39^ This is in line with the findings of the present study, in which some participants who could not prostrate reported that they did not feel the same sense of relaxation.

Although some participants reported difficulty in performing prostration, since they expressed, ‘one cannot feel that they are praying unless they prostrate’, they persisted regardless of the pain. This is consistent with previous reports that found Islamic prayers strengthened a person's relationship with Allah, leading to feelings of security, protection, and calm, which simultaneously reduces pain.^40^ Moreover, some participants explored the effects of prayer and reciting Quranic verses on pain. They used phrases such as ‘powerful medicine’ to describe the analgesic effects that lead to feelings of comfort and peace (*sakina*). Islamic texts also describe the curing effects of Quranic verses, and Allah explains that the Quran is a cure (*shifa* [Shifa means cure from illness]) for all diseases, both physical and spiritual (see the Quranic chapters Yunus [10:57] [Yunus is a Quranic verse], Al‐Isra [17:82] [Al‐Isra is a Quranic verse], and Fussilat [41:44] [Fussilat is a Quranic verse]). Thus, as Muslims believe that Quranic verses have healing effects, it is imperative to consider religious and spiritual aspects when managing pain and suffering.

**Figure 1:**
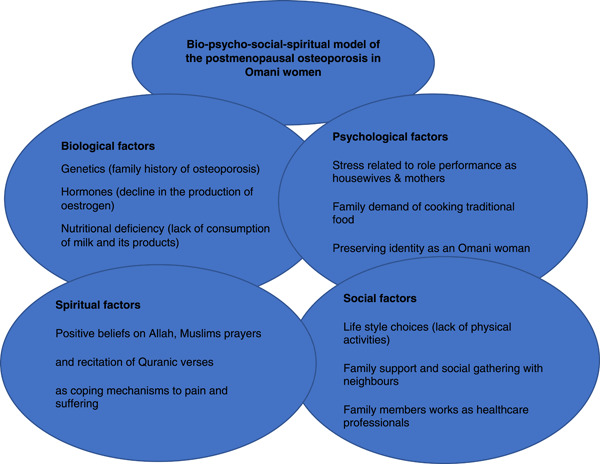
Bio‐psycho‐social‐spiritual model of the postmenopausal osteoporosis in Omani women.

Life‐threatening illnesses can affect a person completely,^41^ not just biologically, psychologically, and socially,^42^ but also spiritually.^43^, ^44^ The biopsychosocial model was proposed by Engel, who stated that ‘all three levels of the life of an individual, biological, psychological, and social, must be taken into account in every health care task’ (p. 170).^12^ The model was expanded to include spiritual factors to fulfil the concepts of holistic healthcare, which address the totality of a patient's relational existence.^45^ The biopsychosocial‐spiritual model suggests that any illness disrupts biological, interpersonal, and spiritual relationships unique to an individual.^46^ This is in line with the findings of the present study, in which osteoporosis and fragility fractures affected the ability of some participants to perform prayer and recite Quranic verses. The biopsychosocial‐spiritual model recognises the potential impact of spiritual and religious variables that may increase or decrease the experience of illness and pain responsiveness.^46^ Figure 1 shows the biopsychosocial‐spiritual model of the impact of postmenopausal osteoporosis in Omani women.

The participants in this study held positive beliefs about their pain and suffering, namely that ‘with more pain, they would be further rewarded by *Allah’*, which is consistent with some Quranic verses. They reported that their positive beliefs led to decreased pain, increased pain tolerance, and positive experiences of their illness. However, some participants also stated that they were unable to tolerate physical religious rituals, especially prayers, because of pain. Given the value of prayer and rituals and attitudes toward pain and suffering in Islam, these contradictions are noteworthy.

Positive beliefs in Quranic verses were linked with pain relief, enhanced pain tolerance, and a more positive outlook towards the diagnoses of osteoporosis and fragility fractures in the study participants. This corresponds with Siddall et al.,^45^ who reported a correlation between religious practices and pain tolerance in patients with chronic illnesses. None of the individuals in this study, regardless of their fragility fracture status, exhibited fear of future fractures. Research indicates that an individual's pain perception may be intensified by fear and anxiety, with fear being responsible for approximately 60% of this effect.^47^, ^48^, ^49^ These data align with the narratives of our participants; their strong spirituality may have alleviated their anxiety and fear concerning their condition, resulting in decreased pain levels.

Engaging in spiritual practices, such as reading Quranic verses or praying, may provide a helpful distraction from pain. Mindfulness‐based interventions, which often involve focusing on the present moment and cultivating nonjudgmental awareness, can be effective in managing chronic pain.^50^ Spiritual practices can offer similar benefits. It is important to note that participants who were unable to recite Quranic verses because of their low literacy levels listened to them instead, which appeared to have similar effects to recitation.

Few American healthcare professionals were found to incorporate discussions about spirituality and religiosity into their patient care, despite many patients wanting these to be considered among their healthcare options.^51^ Moreover, the UK Nursing and Midwifery Council^52^ do not recommend that healthcare professionals discuss religious practices with their patients. However, religious beliefs and practices can be effective in coping with chronic pain management, and these could be included as a holistic approach to patient care, which is in line with the relational models of health that suggest that caring for the sick requires attending to all the patients' disrupted relationships, including biological, neurological, psychological, social, and spiritual.^46^ Thus, healthcare professionals should be aware of the importance of spirituality and religiosity when helping and supporting those experiencing pain.^53^

In addition to the importance of addressing religiosity, cultural beliefs play a significant role in a holistic approach to coping with chronic pain. Some participants believed that herbal remedies could be used as pain‐relief strategies and reported high satisfaction with this therapy, which is consistent with previous studies.^54^, ^55^, ^56^ Further, while some participants used a combination of herbal remedies and Western medicine to manage osteoporotic pain, others relied solely on herbal remedies, possibly owing to their strong faith in herbal remedies, such as traditional Arabic and Islamic medicines.

Although several participants perceived herbal remedies as natural products with no side effects, some herbal remedies have been shown to have serious side effects.^57^ Rivera et al.^58^ highlight that care should be taken when combining herbal remedies with certain Western medicines. The UK Traditional Herbal Medicines Registration (THR) Scheme was introduced by the Medicines and Healthcare Products Regulatory Agency (MHRA) in October 2005 to ensure that these products met specific standards of safety and quality.^59^ More than 320 products now have a THR, and unlicensed products can no longer be sold to consumers.^59^ Although the regulation of herbal medicines continues to be an important safeguard, safety concerns still exist.^59^ To prevent possible contraindications, patients and their relatives should discuss the use of herbal remedies with their healthcare professionals; however, some participants in this study reported never doing so. This lack of communication regarding herbal remedies may be due to a strong cultural faith in such remedies and the belief that natural products do not cause any harm.

Cultural beliefs differed between postmenopausal Omani women and those in a previous review exploring the experiences of patients with osteoporosis.^60^ Barker et al.^60^ set the experience of living with osteoporosis within a cultural framework with certain views regarding osteoporosis as an ageing female condition. In this study, the cultural aspects were underpinned by the use of herbal remedies, the gender role of women, types of physical activities, and cultural reliance on daughters' support.

Osteoporosis and fragility fractures cause massive losses in both productivity and income. The financial effects of osteoporosis led to some participants being dependent on and a burden on their families. This is consistent with the findings of Rocha et al.,^61^ which showed an association between osteoporosis and health‐related productivity loss in postmenopausal Brazilian women. Barker et al.^60^ highlight that the main reason for the financial burden for patients with osteoporosis was job loss. In contrast, all participants in this study who reported financial burden had businesses and thus no end‐of‐service pensions or retirement. The economic burden of osteoporosis and fragility fractures among postmenopausal women in private businesses has not been previously discussed.

### 4.1 Strengths and limitations

To the best of our knowledge, the current study is the first to explore postmenopausal Arab women and their lived experiences with osteoporosis and fragility fractures. These findings provide a basis for future qualitative studies on postmenopausal osteoporosis in this population. Furthermore, the adoption of an interpretative phenomenological approach generated in‐depth data on the impact of cultural and religious practices on the lived experiences of postmenopausal Omani women with osteoporosis. The strength of this study lies in the in‐depth nature of the interviews and openness of the participants' responses, which are integral to an interpretive phenomenological approach. Conducting interviews with women from conservative backgrounds who are influenced by social and cultural standards, gender‐based biases, and stereotypes is of the utmost significance. Providing a secure environment in which women can express themselves without fear of judgement is essential for tackling obstacles in achieving their goals. Empowering these women and striving for equal rights is imperative, ensuring that their voices and contributions are acknowledged.

A limitation of this study is that fewer participants were recruited than planned owing to the COVID‐19 pandemic. Furthermore, the audio‐only interviews limited the observation of facial expressions, which may have provided valuable nonverbal clues.

## 5 CONCLUSION

This study contributes to understanding the experiences of postmenopausal Omani women with osteoporosis and fragility fractures. In contrast to studies on Western women, which found that the provision of health information and regular exercise had an impact on their ability to manage osteoporosis, by focusing on women in a conservative Middle Eastern Islamic country, this study identified the importance of culture and religion on the management of osteoporosis. Given the ageing demographic across the globe,^62^ it is incumbent on policy makers and frontline healthcare professionals to consider the best manner to support chronic illness and long‐term conditions. By highlighting the very different methods of coping with osteoporosis amongst women in Oman, it is hoped this paper can speak to the need for more culturally tailored self‐management techniques across socially and religiously conservative nations throughout the world.

## AUTHOR CONTRIBUTIONS

Faiza Al Zadjali conducted project administration, data collection, data analysis, and wrote the original draft of the manuscript. Jane Brooks provided supervision, provided methodology support, data analysis, reviewed, and edited manuscript drafts. Terence W. O'Neill provided supervision, reviewed, and edited manuscript drafts. Emma Stanmore provided supervision, provided methodology support, reviewed, and edited manuscript drafts. All authors read and approved the final version of the manuscript.

## CONFLICT OF INTEREST STATEMENT

The authors declare no conflict of interest.

## ETHICS STATEMENT

Ethical approval was obtained from the University Research Ethics Committee at the University of Manchester on 30 April 2020 (Ref: 2020–8891) and from the Research Ethical Review and Approval Committee of Oman's Ministry of Health (MoH on 10 June 2020 (Ref: MoH/CSR/20/23534). The consent form was read to all participants, and they agreed to the statement, ‘I agree that any data collected may be published in anonymous form in academic books, reports, or journals’. All participants provided verbal consent to participate in the study.

## Data Availability

The authors confirm that the data supporting the findings of this study are available within the article (and/or) its Supporting Information Materials.
